# A model selection approach to discover age-dependent gene expression patterns using quantile regression models

**DOI:** 10.1186/1471-2164-10-S3-S16

**Published:** 2009-12-03

**Authors:** Joshua WK Ho, Maurizio Stefani, Cristobal G dos Remedios, Michael A Charleston

**Affiliations:** 1 School of Information Technologies, The University of Sydney, NSW 2006, Australia; 2 Muscle Research Unit, Bosch Institute, Discipline of Anatomy and Histology, The University of Sydney, NSW 2006, Australia; 3 Sydney Bioinformatics and Centre for Mathematical Biology, The University of Sydney, NSW 2006, Australia; 4 NICTA, Australian Technology Park, Eveleigh, NSW 2015, Australia

## Abstract

**Background:**

It has been a long-standing biological challenge to understand the molecular regulatory mechanisms behind mammalian ageing. Harnessing the availability of many ageing microarray datasets, a number of studies have shown that it is possible to identify genes that have age-dependent differential expression (DE) or differential variability (DV) patterns. The majority of the studies identify "interesting" genes using a linear regression approach, which is known to perform poorly in the presence of outliers or if the underlying age-dependent pattern is non-linear. Clearly a more robust and flexible approach is needed to identify genes with various age-dependent gene expression patterns.

**Results:**

Here we present a novel model selection approach to discover genes with linear or non-linear age-dependent gene expression patterns from microarray data. To identify DE genes, our method fits three quantile regression models (constant, linear and piecewise linear models) to the expression profile of each gene, and selects the least complex model that best fits the available data. Similarly, DV genes are identified by fitting and comparing two quantile regression models (non-DV and the DV models) to the expression profile of each gene. We show that our approach is much more robust than the standard linear regression approach in discovering age-dependent patterns. We also applied our approach to analyze two human brain ageing datasets and found many biologically interesting gene expression patterns, including some very interesting DV patterns, that have been overlooked in the original studies. Furthermore, we propose that our model selection approach can be extended to discover DE and DV genes from microarray datasets with discrete class labels, by considering different quantile regression models.

**Conclusion:**

In this paper, we present a novel application of quantile regression models to identify genes that have interesting linear or non-linear age-dependent expression patterns. One important contribution of this paper is to introduce a model selection approach to DE and DV gene identification, which is most commonly tackled by null hypothesis testing approaches. We show that our approach is robust in analyzing real and simulated datasets. We believe that our approach is applicable in many ageing or time-series data analysis tasks.

## Background

### Age-dependent gene expression patterns discovery in microarray datasets

Ageing is an important risk factor to many diseases, but the molecular basis of this complex process is still poorly understood [1]. Due to the advances in high-throughput experimental technologies, an increasing number of large-scale microarray studies have been conducted to identify ageing associated genes in human and model organisms [2-7]. There are two important types of age-dependent gene expression patterns that are of particular interest to the community: differential expression (DE) patterns, and differential variability (DV) patterns. A gene is said to have age-dependent DE if its expression has a strong positive or negative correlation with ageing. Similarly, a gene has age-dependent DV (also called age-dependent variability or heterogeneity [8,9]) if it exhibits a strong increase or decrease of expression variability (or heterogeneity) with ageing.

The identification of genes with age-dependent DE patterns is the central microarray analysis task of many ageing studies. For instance, linear regression is the principle tool for identifying genes with strong (linear) age-dependent expression trends in two recent large meta-analysis of ageing microarray studies [5,7]. Linear regression is a statistical method that models a dependent variable (usually denoted as *y*) as a linear function of one or more independent variables (usually denoted as *x*). The linear function takes the form *f*(*x*, *θ*) = *a *+ *bx *where *θ *= {*a*, *b*}; therefore solving the linear regression problem is equivalent to estimating the parameter vector, *θ*. In the context of age-dependent gene expression pattern discovery, *y *is the expression of a gene, and *x *is age. Given the expression profile of a gene in the form of , the parameter vector *θ *can be estimated by the method of ordinary least squares, which can be written as the following minimization problem:

The estimated linear function  is an estimate of a conditional mean function of the data. Once the linear regression function is estimated, a *p*-value is calculated to determine whether the slope parameter, *b*, is significantly different from zero. If a gene has an associated *p*-value less than a predefined significance level after correcting for multiple testing, this gene is deemed to be *differentially expressed*.

We have previously introduced the concept of differential variability analysis (DVA) and showed that changes in gene expression variability are biologically relevant in understanding human diseases [10]. Our approach is based on a trimmed *F*-test on two groups of samples (e.g., disease vs. non-disease). One major limitation of our previous approach is that we are restricted to analyzing microarray datasets in which samples are grouped into discrete classes. This limitation excludes the application of our DVA method to discover age-dependent DV genes. However, it is evident that such age-dependent variability changes are real and biologically relevant. Bahar *et al*. [11] showed that there is an increase in cell-to-cell gene expression variation in aged mice's heart muscle compared to those of younger mice. Somel *et al*. [8] showed that there are a statistically significant number of genes that have increased variability (or heteroskedasticity) in ageing by re-analyzing eight microarray datasets for human and rat. Such an age-dependent increase in gene expression variability is also supported by a recent experiment that was designed particularly for studying gene expression variability changes in rat retina [9], which have identified 340 genes with significant increase in expression variability across ages, but only 12 genes with significantly decreased expression variability [9]. Many of these genes are found to be biologically relevant to the process of ageing. The analysis method used in both studies relied on a two step procedure: (1) Obtain residuals of the expression value after fitting a regression model (or an ANOVA model) for every gene, and (2) Determine whether there is a statistically significant change in variability across age by fitting another linear regression model through the absolute values of the residuals.

Despite the wealth of microarray time-series analysis procedures devised to date (such as [12,13]), only simple linear regression methods are used in analyzing microarray data generated from most of the published ageing studies (for example, [3,5,7-9]). We believe this is due to the nature of the common experimental designs of this type of ageing study, which precludes the need for mining more complex time-series patterns (such as oscillation of gene expression). Ageing studies are typically designed to look at age-dependent steady state gene expression changes at a population level, therefore the fine-grained dynamic molecular responses of a cell to particular external or internal stimuli is not of great concern. Despite many recent studies showing that accurate identification of genes with age-dependent DE and DV patterns can lead to deeper biological insight into the complex regulatory processes through ageing [5,7-9], relatively little attention has been paid to the bioinformatics methods of identifying such patterns. Since linear regression approach is known to perform poorly in the presence of outliers or if the underlying pattern is non-linear, we sought a more robust and flexible method to identify various age-dependent patterns. In this paper, we present a simple solution based on the technique of *quantile regression*. The basics of quantile regression are introduced in the next subsection, followed by a detailed description of our new approach in the **Results **section.

### Introduction to quantile regression

The standard linear regression approach aims to estimate a conditional mean function of  = *f*(*x*) given any *x*. Quantile regression, on the other hand, aims to estimate a conditional quantile function for any quantile 0 <*τ *< 1. For instance, we can obtain a conditional median function by estimating a quantile regression function with *τ *= 0.5. In addition to its robustness against outliers, quantile regression gives flexibility in terms of modeling various parts of a data distribution beside the mean [14].

The quantile regression technique was first developed by Koenker and colleagues in 1978 [15] and has been continuously studied and extended since then [14]. It has been used in various fields such as econometrics [16] and ecology [17,18]. Quantile regression has also been recently applied to various areas of bioinformatics, such as visualization of array Comparative Genomic Hybridization (CGH) data [19,20], identification of differentially expressed genes in two-color microarray datasets [21] and outlier detection in mass spectrometry data [22].

Similar to the formulation of linear regression, the aim of quantile regression is to estimate the parameter vector, *θ*, of a quantile function *y *= *f*(*x*, *θ*) given a data series . The main difference between quantile regression and linear regression is that *θ *is estimated by minimizing an objective function based on a skewed absolute difference between every *y*_*i *_and *f *(*x*_*i*_, *θ*), as shown below:

where *ρ*_*τ *_(*u*) is a *check function *(also called *pinball function*) with parameter *τ *which specifies the quantile.

The check function is defined as:

We can obtain various linear and non-linear quantile regression lines by using different parametric models for the quantile function. In this paper, we refer to such a parametric model as a *quantile regression model*. We focus on three basic quantile regression models in this paper: the constant model, the linear model and the piecewise linear model. The constant model takes the form *f*_*c*_(*x*, *θ*_*c*_) = *a *where *θ*_*c *_= {*a*}. The linear model takes the form *f*_*l*_(*x*, *θ*_*l*_) = *a *+ *bx *where *θ*_*l *_= {*a*, *b*}. The piecewise linear model takes the form

where *x*_0 _is the location of the change point and *θ*_*pl *_= {*a*, *b*_1_, *b*_2_, *x*_0_}. We note that our piecewise linear model specifies a continuous piecewise linear function with one change-point at (*x*_0_, *a *+ *b*_1_*x*_0_). These three models form the basis of our approach for identifying various age-dependent gene expression patterns.

## Results

### Our approach

Here we describe our novel method to discover various age-dependent gene expression patterns based on a model selection strategy. An important observation is that the goodness-of-fit of a quantile regression model to a given data series can be assessed by the *residual sum of absolute differences *(RSAD), which is analogous to the residual sum of squares (RSS) in the linear regression case. Given the estimated parameter of a quantile regression model as in Equation 2, RSAD is defined as

In other words, RSAD is the optimal value of the objective function after solving the minimization of Equation 2. The smaller the RSAD, the better a model fits the data. It is also known that a model with more parameters tends to gives lower RSAD than a model with fewer parameters (see [23] for a discussion). For example, the RSAD of fitting a linear quantile regression model must be smaller than or equal to the RSAD of fitting a constant quantile regression model to the same data series. The RSADs of the fitted constant and linear models are the same when the a parameter in both models is the same, and parameter *b *of the linear model is zero, since other non-zero estimate of *b *should always give a smaller RSAD for the linear model. The main idea of our approach is to select the least complex model which can fit the data with a low RSAD. In the context of model selection, a model *M*_1 _is more complex than *M*_2 _if *M*_1 _has more parameters than does *M*_2_. Therefore, we can order our three quantile regression models from the least complex to the most complex as: constant, linear, and piecewise linear. We note that the three models are *nested *in the sense that a less complex model can be obtained by imposing constraints to a more complex model (that is, a model with more parameters). A piecewise linear model with a constraint *b*_1 _= *b*_2 _is identical to a linear model regardless of the parameter choice of *x*_0_, and a linear model can be reduced to a constant model by restricting *b *= 0 in the linear model. Various criteria can be used for model selection, including various information-theoretic criteria [23]. In this paper, we present a simple, yet intuitive, criterion for choosing between two quantile regression models: select a more complex model over a more simple model if the ratio of the RSADs of the two fitting models is smaller than a predefined threshold. The optimal threshold of a particular problem can be chosen by considering the estimated false discovery rate at different threshold values, which is further explained later in the paper.

**Figure 1:**
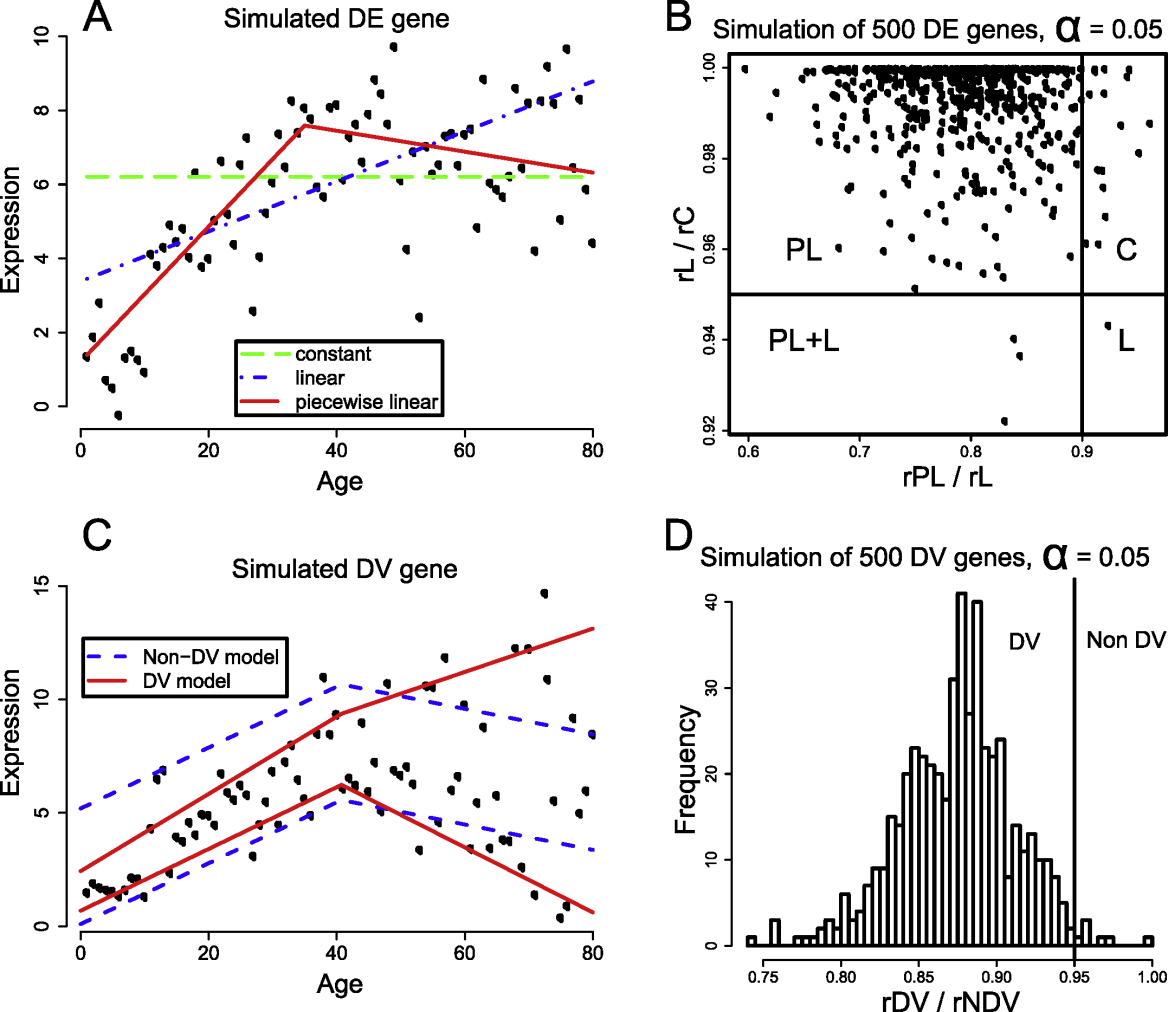
**Illustration of our model selection approach to identifying both age-dependent DE and DV patterns**. The four plots in this figure illustrate the core idea of our model selection approach to identifying genes with age-dependent change in expression (DE) or variability (DV). (A) A gene with an artificially simulated expression profile is fitted with three quantile regression models: the constant model, the linear model and the piecewise linear model. The estimated quantile regression lines are superimposed onto the expression profile. The simplest model that fits the data reasonably well is selected to be the target model. If the linear model or piecewise linear model is selected, this gene is said to be DE. (B) The distribution of *rPL*/*rL *and *rL/rC *of 500 artificially simulated genes with non-linear age-dependent expression changes. Given a predefined threshold, a, all genes are partitioned into one of four groups (C, L, PL, and PL+L), based on where they are located in this plot. (C) This plot shows the expression profile of a gene that has been simulated to have increasing variability with ageing. Both a non-DV and a DV quantile regression models are fitted to the data of this gene, and the fitting regression quantile lines are superimposed onto the plot of the expression profile. (D) A histogram showing the distribution of the *rDV/rNDV *value generated by fitting the non-DV and DV models to 500 simulated genes with DV. Most of the genes have a *rDV/rNDV *value less than the (1 - *α*) threshold, and are therefore correctly identified as DV.

To determine if a gene exhibits a DE pattern, we separately fit to the expression profile of that gene three quantile regression models at *τ *= 0.5: the constant model, the linear model and the piecewise linear model with one change-point as presented in the **Background **section. The model that best describes the available data is said to be the target model of the gene (see Figure 1A for an example of fitting the three models to a gene with a non-linear age-dependent DE pattern). If a linear model or a piecewise linear model is the best fitting model based on a predefined threshold, this gene is said to have an age-dependent DE pattern. Denoting the RSAD of fitting a data series with the constant, linear and piecewise linear models as *rC*, *rL *and *rPL *respectively, we can choose the appropriate model by considering the two ratios: *rPL/rL *and *rL/rC*. We note that both of these quantities must be less than or equal to one, and that the smaller the quantities, the stronger support there is for the more complex model. Based on a predefined threshold *α*, we can select the best fitting model by the following rules (see Figure 1B):

*rPL*/*rL *≥ (1 - 2*α*) and *rL*/*rC *≥ (1 - *α*) ⇒ no DE pattern (C)

*rPL*/*rL *≥ (1 - 2*α*) and *rL*/*rC *< (1 - *α*) ⇒ linear DE pattern (L)

*rPL*/*rL *< (1 - 2*α*) and *rL*/*rC *≥ (1 - *α*) ⇒ piecewise linear DE pattern (PL)

*rPL*/*rL *< (1 - 2*α*) and *rL*/*rC *< (1 - *α*) ⇒ piecewise linear DE pattern with a linear trend (PL+L)

The constant 2 in (1 - 2*α*) arises from the ratio of the number model parameters in each model pair: 4:2 for comparing between a piecewise linear model and a linear model, and 2:1 for comparing between a linear model and a constant model. In general *α *can be chosen based on false discovery rate estimation or by simulation of data. It is important to note that the selection threshold *α *is not a significance level, as is commonly used in the context of null hypothesis testing. The significance level in the null hypothesis testing framework has a probabilistic meaning, while the threshold we used here is to define how much better a more complex model needs to fit the data in order for it to be selected over the simpler model. Similarly we can determine whether a gene has a DV pattern by fitting and comparing the goodness-of-fit of two quantile regression models: the non-DV model and the DV model (Figure 1C). The non-DV model consists of two piecewise linear functions, one for an upper quantile and one for a lower quantile, which share the same slope parameters *b*_1 _and *b*_2 _and change-point parameter *x*_0_. The DV model consists of two piecewise linear quantile regression functions that have independent slope parameters but the same change-point parameter *x*_0_. In both non-DV and DV models, we fit the upper quantile and lower quantile trend model at *τ*_*upper *_= 0.85 and *τ*_*lower *_= 0.15 respectively. We observe that choosing other reasonable values of *τ *(that is, ± 0.1 on both *τ*_*upper *_and *τ*_*lower*_) does not make a substantial difference in practice. The parameters of both non-DV and DV models are estimated by solving a joint optimization problem which can be formulated as follows:

where *θ *= *θ*_*upper *_∪ *θ*_*lower*_. Analogously, the RSAD of both models is the optimal value of the objective function after solving the minimization problem in Equation 6. Using the RSADs of the fitted non-DV and DV models, denoted *rNDV *and *rDV *respectively and a predefined threshold, 0 <*α *< 1, we can determine whether the DV model should be chosen over the simpler non-DV model by checking whether *rDV/rNDV *< (1 - *α*) (Figure 1D).

We use the Broyden-Fletcher-Goldfarb-Shanno (BFGS) method implemented in R's optim function to solve the optimization problems associated with estimating the quantile regression model parameters. BFGS method is a general method to solve unconstrained nonlinear optimization problems.

### Simulation results

We performed an extensive simulation study to empirically establish the sensitivity and specificity of our quantile regression based methods compared with the linear regression based methods (see **Methods**).

The basic experimental design is to simulate datasets with different noise characteristics, and calculate the true positive (TP), true negative (TN), false positive (FP) and false negative (FN) rates in each simulated dataset at different *α *threshold values by checking whether a gene with true age-dependency is correctly identified or not. Further details of the simulation study are given in the **Methods **section. The trade-off between the true positive rates and the false positive rates of a method at different values of *α *is visualized in a Receiver Operator Characteristic (ROC) curve for each simulated dataset.

To test the ability of our method to identify age-dependent DE genes, we simulated five 3000-gene datasets, each containing a different degree and type of noise. There are two types of noise that we investigated here: systematic noise (a consistent amount of noise that affects all the samples regardless of age), and non-systematic outliers (noise that are only present in some data points, which we refer to as outliers). Each simulated dataset consists of three equal proportions of non-DE genes, DE genes with linear age-dependency, and DE genes with non-linear age-dependency. As a base-line, we compared our method with a method based on a second order linear regression method.

**Figure 2:**
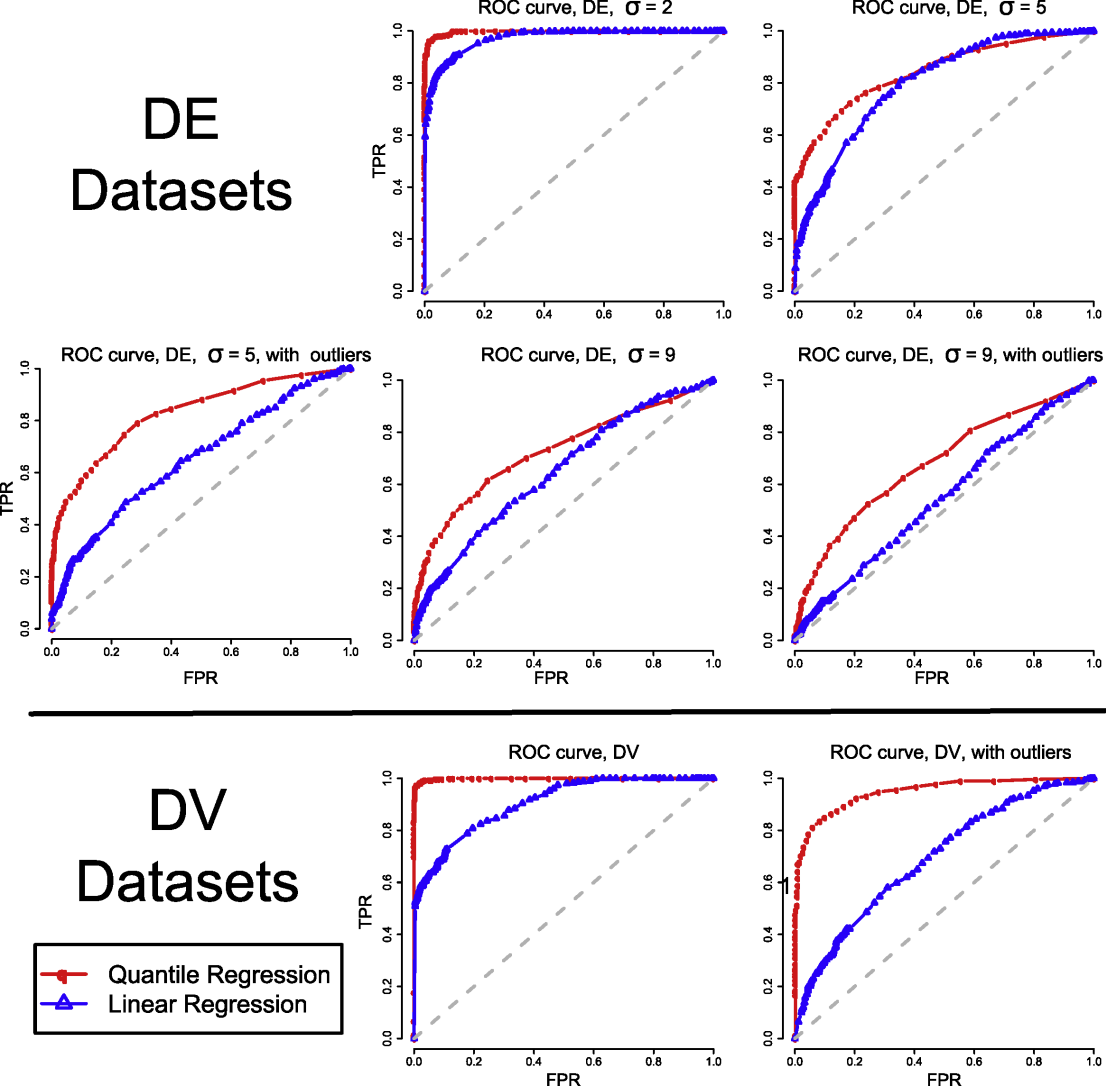
**Comparison of our quantile regression method to a linear regression based method using the ROC curves**. This figure shows the ROC curves generated by analyzing seven simulated datasets using our quantile regression method, and a linear regression method. Each simulated dataset has a different type and level of noise. The ROC curves show that our approach consistently out-performs the linear regression method studied in this work in terms of both sensitivity and specificity in all seven simulated datasets.

**Figure 3:**
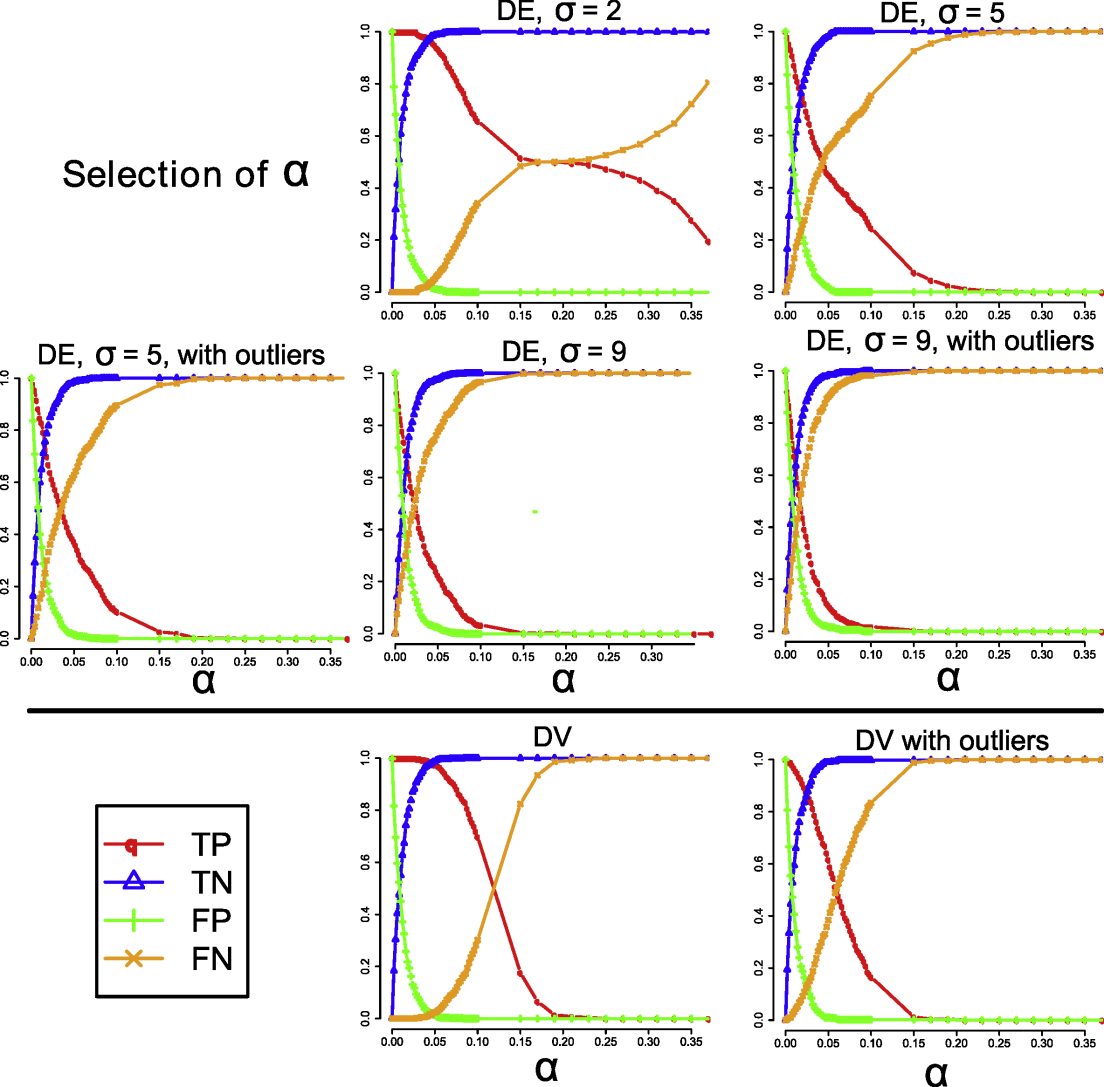
**The relationship of the model selection threshold, *α*, with the various performance measures**. This figure shows how four different performance measures vary with different values of model selection threshold, *α*, based on analyzing the seven simulated datasets The four performance measures are the true positive rate (TP), the true negative rate (TN), the false positive rate (FP) and the false negative rate (FN). We note that an a value between 0.02 and 0.05 generally gives a reasonable trade-off between the true positive rate and the false positive rate. a high true positive rate while maintaining a low false positive rate.

To test the ability of our method to identify age-dependent DV genes, we simulated two 3000-gene datasets, each containing a different type of noise — one dataset without outliers and one dataset with outliers. Each simulated dataset comprised three equal proportions of non-DV genes, DV genes with linear age-dependency, and DV genes with non-linear age-dependency. We compared the performance of our method with a variant of the linear regression based approach of [8] to identify age-dependent variability. The results are summarized in Figure 2. From the ROC curves, we can see that our approach consistently out-performs linear regression based methods in terms of both sensitivity and specificity for both DE and DV detection, regardless of the type and level of noise that is present in the datasets we studied here. One important question is 'how to select the best *α *threshold value?' To address this question, we investigated how TF, TN, FP and FN vary with *α *in our seven simulated datasets. As illustrated in Figure 3, we found that an *α *value between 0.02 and 0.05 is appropriate as it generally shows a good trade-off between sensitivity and false positive rate in our seven simulated datasets. Furthermore, we calculated the false discovery rate (FDR) of each method for the seven simulated datasets at the threshold value 0.05. In this simulation analysis, a false discovery rate is defined as the proportion of false positive calls in all positive calls, i.e., FP/(FP+TP). The results in Table 1 indicate that our quantile regression approach consistently yields FDRs that are only one third of their corresponding FDR of the linear regression based method.

**Table 1:** Comparison of false discovery rate (FDR) of our quantile regression methods and linear regression methods using simulation data.

	DE					DV	
FDR	DE2	DE5	DE5 + outliers	DE9	DE9 + outliers	DV	DV + outliers
Quantile Regression (*QR*)	0.021	0.040	0.049	0.082	0.151	0.017	0.023
Linear Regression (*LR*)	0.061	0.160	0.204	0.230	0.38	0.083	0.262

FDR_*QR*_/FDR_*LR*_	0.340	0.247	0.237	0.357	0.396	0.214	0.087

The FDRs of applying our quantile regression method to seven simulated datasets are compared to the corresponding FDRs of applying linear regression based methods to identify DE and DV genes at a predefined threshold of *α *= 0.05 (for quantile regression) and *α*_*l *_= 0.05 (for linear regression). At this commonly accepted threshold, we found that our quantile regression method yields FDRs that are consistently about only one third of that the corresponding FDR when the linear regression approach is used.

### Analysis of two human brain ageing datasets

We applied our method to analyze two real microarray datasets that study human brain ageing in non-diseased individuals. The Colantuoni dataset [6] consists of gene expression measurements for 31 schizophrenia susceptibility genes in the prefrontal cortex of 72 non-diseased individuals with age range of 18 to 67. The second dataset, which we referred to as the Lu dataset, consists of the expression profiles of 12625 genes for 30 non-diseased individuals with age ranging from 26 to 106 [2].

**Figure 4:**
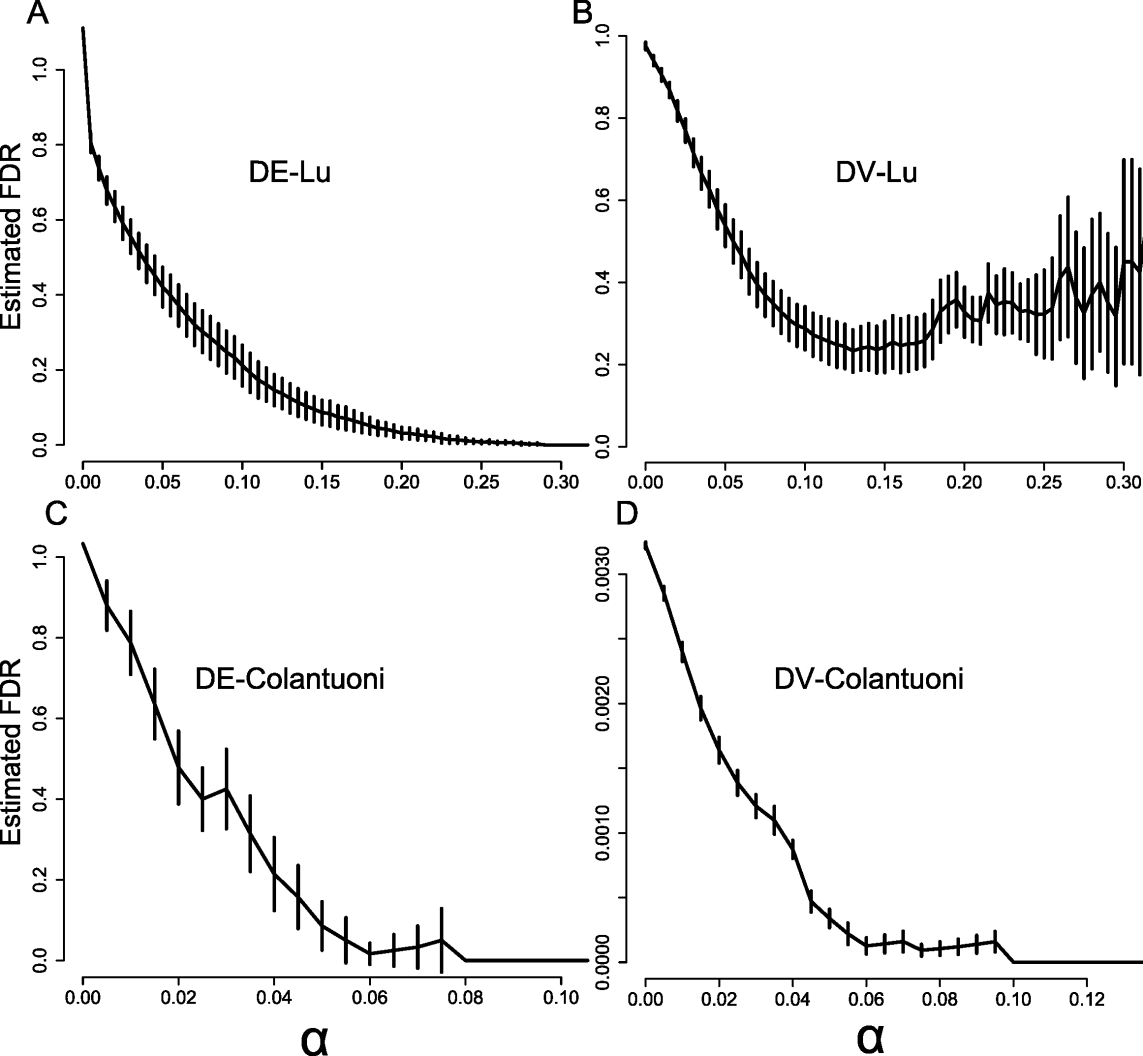
**Estimated FDR at various *α *values for applying our method to the two real datasets**. The means and standard deviations of the estimated FDR of applying our method to the two real datasets. These plots enable us to determine a reasonable *α *value such that the FDR of a real analysis is kept reasonably low.

The false discovery rates of discovering genes with DE and DV patterns at various *α *values were estimated by a randomization procedure that is described in the **Methods **section. The results are shown in Figure 4. To ensure that our DE gene discovery approach yields a low FDR, we chose *α *= 0.04 (at FDR ≈ 0.2) for the Colantuoni dataset and *α *= 0.1 (at FDR ≈ 0.2) for the Lu dataset. For DV gene discovery, we chose *α *= 0.05 (at FDR ≈ 0.0005) for the Colantuoni dataset and *α *= 0.15 (at FDR ≈ 0.2) for the Lu dataset. The analysis was performed on a desktop machine with an Intel Core 2 CPU (1.86 GHz) and a Windows XP (Professional) operating system. The DE analysis of the Colantuoni datasets (31 genes) completed in one second, while the analysis of the Lu dataset (12625 genes) took about 6.5 minutes. The computational time taken to perform the DV analysis for the two datasets is similar.

#### The Colantuoni dataset

**Figure 5:**
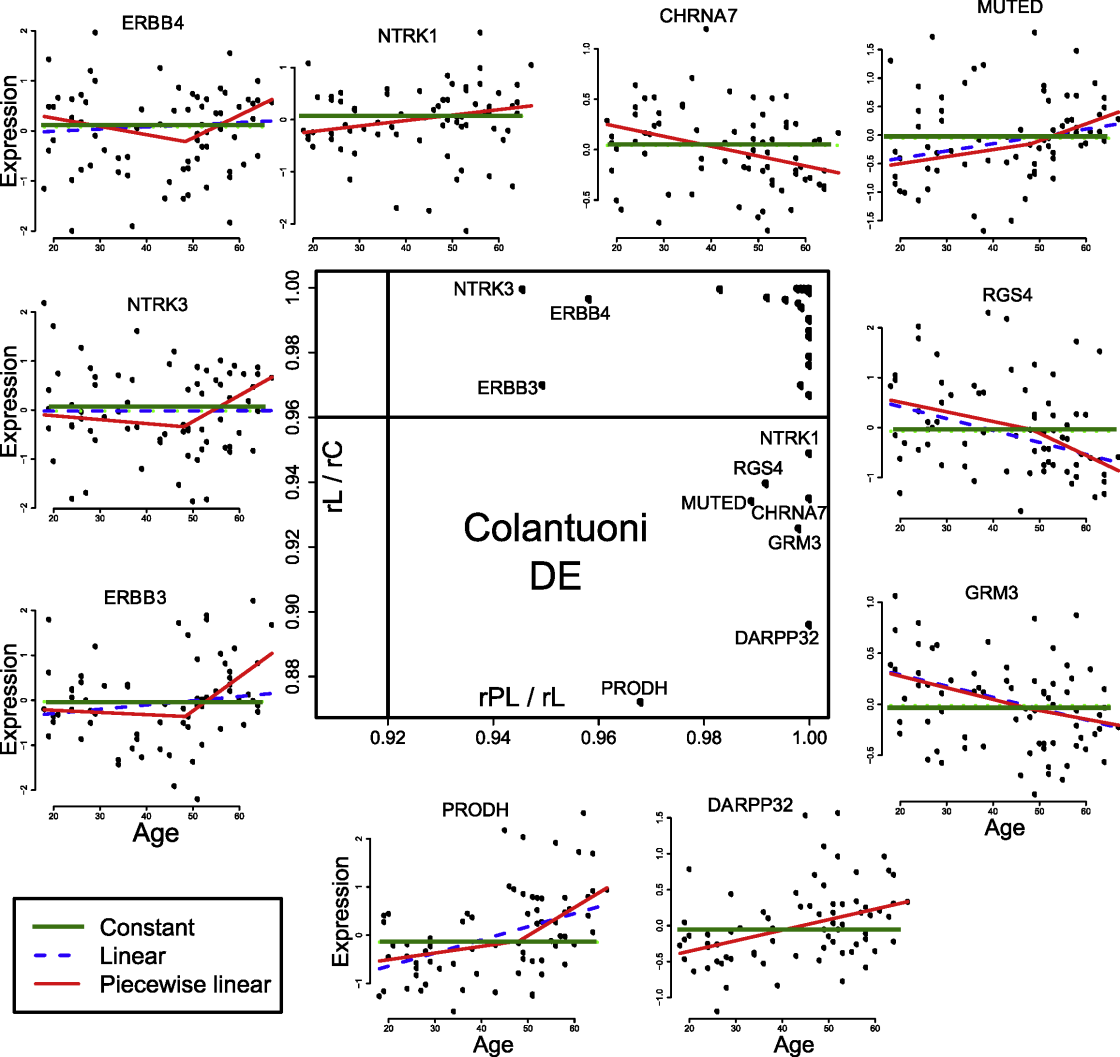
**Some age-dependent DE genes discovered in the Colantuoni dataset**. The plot in the centre of the figure shows the distribution of the *rL/rC *and *rPL/rL *of the 31 genes profiled in the Colantuoni dataset. Based on this plot, seven genes exhibit strong support for a linear age-dependent DE pattern, and three genes have moderate support for a non-linear age-dependent DE pattern. The expression profile of these 10 genes, along with their three fitted quantile regression lines.

**Figure 6:**
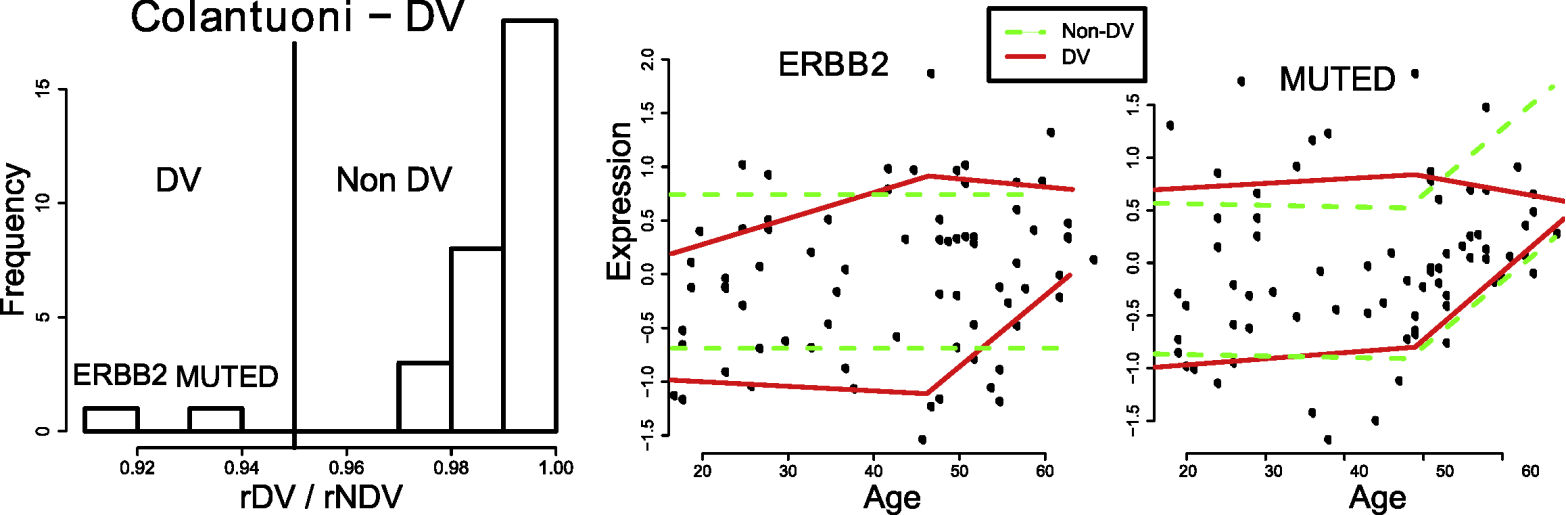
**Some age-dependent DV genes discovered in the Colantuoni dataset**. Based on the *rDV/rNDV *value, we identified two genes, ERBB2 and MUTED, with strong evidence of DV pattern. The expression profile of these two genes shows that the expression variability decreases for both.

Among the 31 genes surveyed in the Colantuoni dataset, we identified ten "interesting" genes, which include seven genes with strong evidence for the presence of a linear DE pattern (PRODH, DARPP32, GRM3, CHRNA7, MUTED, RGS4 and NTRK1), and three genes with a moderate support for a non-linear DE pattern (NTK3, ERBB3, and ERBB4). A plot showing the *rL/rC *and *rPL/rL *values for all the genes, along with the expression profiles of these 10 genes, is given in Figure 5. Independently, we used our method to discover two genes with strong support for DV (ERBB4 and MUTED; see Figure 6). Most of our results are consistent with what was found in the original study [6], but our analysis reveals three major differences.

First, by fitting a piecewise linear regression function (with one change-point) to all genes Colantuoni *et al*. identified three genes (ERBB3, NRG1 and NGFR) to have "statistically significant" changes in the slope of the two segments of the linear regression line about the change-point. However, among the three, only ERBB3 has a reasonably good support for having a non-linear DE pattern in our analysis based on its low *rPL*/*rL *value (see Figure 5). Instead, we found good evidence that NTK3 and ERBB4 exhibit such a piecewise linear DE pattern since their *rPL/rL *values are low and cluster quite closely with ERBB3 in our plot of *rPL/rL *against *rL/rC *(Figure 5). Further we note that although no gene actually has a *rPL/rL *value less than (1 - 2*α*), the fact that the *rPL/rL *values for these three genes are much lower from the rest of the 28 genes already implies that these genes have some kind of interesting patterns, and should be investigated further.

Second, MUTED is determined to not have a significant linear correlation with age because its associated *p*-value (0.062) is just a little higher than the predefined threshold (0.05), so no further analysis on this gene was undertaken. However, our analysis shows that MUTED exhibits both age-dependent DE and DV patterns, which warrant further investigation. The expression of MUTED increases with age, but the variability decreases. MUTED codes for a component in the BLOC-1 complex, which is involved in the trafficking of particular membrane proteins to synaptic vesicles during their formation [24]. It is uncertain what effect an increase in MUTED expression will have, however decreased variability of expression might reflect a loss of regulation of MUTED activity. This might manifest as decreased synaptic plasticity with age occurring at the level of synaptic vesicle maturation.

Third, ERRB2 is found to have the strongest support to exhibit an age-dependent DV pattern (Figure 6), but it is deemed "not significant" as it only has a *p*-value of 0.056 found in the original study [6]. ERRB2 is a member of the epidermal growth factor receptor family (ErbB) of receptor tyrosine kinases. It is expressed in neurons in the adult cerebral cortex and hippocampus and on oligodendrocytes, and is involved in neuronal migration/glial cell-neuron interactions during CNS development, as well as oligodendrocyte maturation/myelination [25]. We found that the variability of ERBB2 expression also decreases with age (see Figure 6). A hypothesis of such changes of expression variability is that the aged prefrontal cortex is attempting to compensate for structural or functional deficits by *de novo *neurogenesis, neuronal migration or myelination which requires upregulating ERBB2 expression (and some brains are more efficient at carrying out this compensatory process), but that there is a ceiling of maximum upregulation, and this is resulting in the phenomenon of decreased DV. Indeed, it has been suggested that the prefrontal cortices increase their activity/connectivity with age in response to declining cognitive function in other areas of the brain [26]. Further experimental validation is needed to test our hypothesis, but our analysis here is sufficient to show that our quantile regression approach is useful for identifying genes with interesting DV patterns. Also, the quantile regression lines themselves act as a good tool for visualizing the DV patterns, which aid the interpretation of the results.

#### The Lu dataset

**Figure 7:**
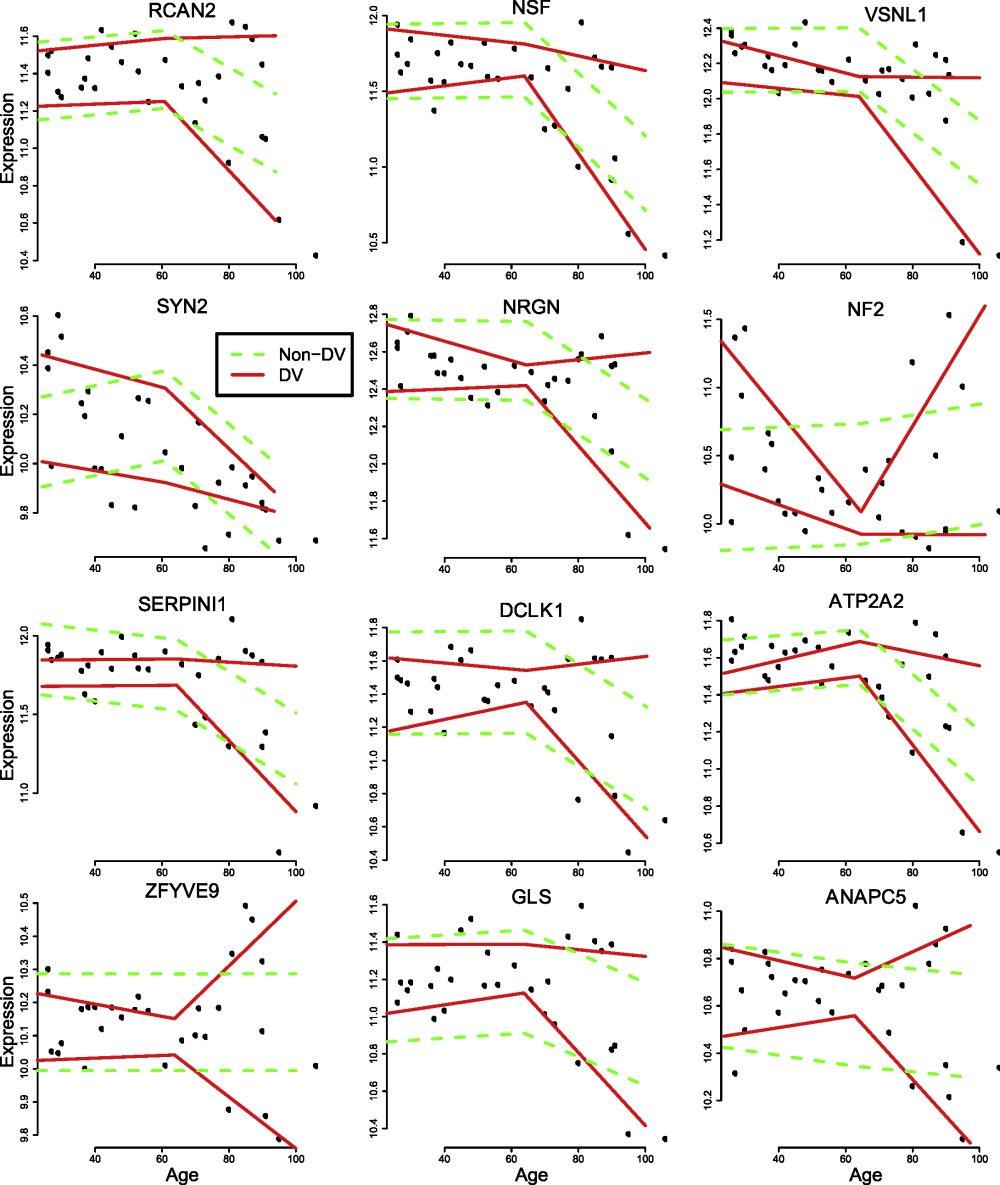
**Some age-dependent DV genes discovered in the Lu dataset**. This figure shows 12 genes with strong evidence of DV (by having small *rDV/rNDV *value) in the Lu dataset. Most of these genes have an increasing expression variability among older individuals. Many of these genes are found to have be involved in important neuronal processes.

By applying our quantile regression approach to analyze the Lu dataset, we found 984 genes with linear DE pattern, 12 genes with non-linear DE pattern, and 120 genes with DV pattern. Since most of the genes we found with strong evidence of DE are also found and analyzed in the original study, we mainly focused on analyzing the genes that show strong evidence of DV. The expression profiles of 12 selected genes with strong evidence of DV, i.e., having a low *rDV*/*rNDV *ratio, are shown in Figure 7. We observed that most of these 12 genes exhibit increasing expression variability with age (including RCAN2,, NGRN, SERCA2, NSF, SERPINI1), and this change in variability seems to correlate with a reduction in expression of varying magnitudes. This implies differing trajectories of ageing for different individual brains. Calcineurin is a serine-threonine kinase, abundant in the brain, that regulates neuronal cell death, neurite outgrowth and synaptic plasticity. RCAN2 (Regulator of calcinuerin 2) is a facilitatory regulator of calcineurin activity. It was shown that RCAN1/2 double knock-out (KO) mice exhibit hyperactivity and working memory deficits [27]. NRGN (Neurogranin) is expressed in the forebrain and hippocampus and is a regulator of Ca^2+^-mediated and Ca^2+^-CaM-mediated signalling pathways. NGRN mRNA and protein expression has been shown to decrease with age [28,29]. NRGN KO mice show deficits in spatial learning [30,31] with associated disrupted CaMKII activity and LTP [30,32]. The gene ATP2A2 codes for the sarco/endoplasmic reticulum Ca^2+ ^ATPase pump 2 (SERCA2), which is highly expressed in various parts of the brain, including the hippocampus and cortex [33]. It is involved in regulating intracellular Ca^2+ ^homeostasis. NSF (N-ethylamide sensitive factor) is a key protein associated with a myriad of processes in the central nervous system including trafficking of synaptic vesicles and regulation of neuronal glutamatergic, GABAergic, adrenergic and muscarinic membrane receptors [34]. SERPINI1 codes for a protein involved in many processes including synaptic plasticity and the prevention of neuronal death due to ischemia. A number of mutations of SERPINI1 are shown to be associated with early-onset dementia [35]. The pattern of increased DV of these genes with important roles in neuronal function and pathology with varying reduction in expression with age implies a possible role in the observed differential rate/incidence of cognitive decline in older people [26]. It seems that of all the genes found to be DV with age, this pattern is the most common, implying that loss of maintenance of stable expression of genes expressed in the central nervous system might be a key process in ageing.

Since Lu *et al*. [2] used a microarray platform that interrogated the entire genome, as opposed to a brain specific platform used in Colantuoni *et al*. [6], the ability of our method to identify many DV genes to have known roles in various neurological processes further reinforces the importance of identifying DV genes when performing microarray analysis.

## Discussion

### Some remarks on our approach

Our approach is based on selecting the least complex model that is reasonably strongly supported by the data. There are two important ingredients that need to be defined in a model selection approach: (1) A small set of models which we believe are able to explain the data, and (2) A set of criteria that enables us to compare these models. In this work, we show how the task of identifying age-dependent gene expression patterns from ageing microarray datasets can be formulated as a model selection problem and solved accordingly.

This model selection approach to scientific data analysis is strongly advocated by Burnham and Anderson [23], who note that the original concept was proposed more than a century ago by Chamberlin under the name of *method of multiple working hypotheses *[36]. Although Chamberlin's work mostly focuses on the philosophy of scientific investigation, that multiple working hypotheses should be considered simultaneously when designing a research study, the work by Burnham and Anderson focuses on the use of model selection as a preferred method over null hypothesis testing approach when analyzing and interpreting scientific data. In this sense, the method that we present in this paper is indeed an application of the concept of model selection to solve a problem that is commonly solved by null hypothesis testing approaches.

Similar to a null hypothesis approach, our approach also requires a threshold, which we referred to as *α*, to be defined prior to the analysis in order to decide if a gene is deemed to have "interesting" patterns, either DE or DV. The threshold *α *controls the minimum proportional difference in RSAD, i.e., (RSAD_*S *_- RSAD_*C*_)/RSAD_*S*_, in order for a more complex model (M_*C*_) to be selected over a less complex model (M_*S*_). In general, we believe such a model selection threshold is intuitive and easily extended to analyzing more complex models since no null distribution has to be defined. Further, we note that the term "significant" or "significance" were not used to describe a gene we identified to have strong support for a particular pattern, as these wording tend to be misleading. Moreover we note that our approach is similar to the likelihood ratio test method if we treat RSAD to be inversely related to the likelihood of fitting a model. A further research direction is to investigate how a model selection strategy based on information theoretic criteria such as Akaike Information Criteria (AIC) or Bayesian Information Criteria (BIC) is compared to our approach.

Our application of quantile regression in analyzing ageing microarray datasets has three advantages over the standard linear regression method in analyzing microarray time-series data — robustness against noise, ease of visualizing DV patterns, and the ability to model various parts of a data distribution — which are all clearly exemplified in our analysis of the simulated and real datasets. In particular, we stress the importance of obtaining a regression trend at various quantiles, rather than a regression trend through the mean of a distribution. It has been argued that a biologically important limiting factor in ecological studies may not affect the average behaviour of the measured variable, but may strongly affect the behaviour at the extreme quantiles [17]. Such a phenomenon is attributed to the effect of unobserved variables.

Another contribution of our paper is the application of a piecewise linear quantile regression model to identify genes with age-dependent DE and DV patterns. The application of piecewise linear regression for biological responses has been studied by [37,38]. Here we explicitly use a piecewise linear quantile regression model with one change-point. We chose to use a piecewise linear model to model various gene expression patterns because it is a flexible yet interpretable model. Non-linear or non-parametric model can provide similar level of flexibility but is hard to interpret without manual inspection. For high-throughput data analysis, being able to uncover a wide range of patterns without manual intervention is highly desirable. The change-point location may also be biologically informative.

From a methodological point of view, our work still has a few limitations. First, although we have empirically validated the superior performance of our approach in analyzing noisy microarray data, we did not give any theoretical justification of why this is the case. Without further investigation it is very difficult to discern how much of this improvement is due to the model selection strategy, and how much is due to the robustness of the quantile regression method. This should therefore be further investigated. Second, we only used a generic non-linear optimization algorithm to solve our optimization problem (as in Equations 5 and 6) associated with estimating the parameters of a quantile regression model. Although the BFGS method works well in practice given a good initial parameter estimates, there is no guarantee that the result is indeed the global optimum. This is an even larger problem with models that have many parameters as they are more likely to have complicated (e.g., non-convex) solution surfaces. One line of research direction is to re-frame the optimization problem as a linear programming problem and solve it with the Simplex method [14].

### Biological significance of differential gene expression variability in ageing

A number of recent studies showed that the changes in expression variability may be associated with mammalian ageing [8,9] and human diseases [10]. In this paper, we provide further evidence that differential gene expression variability is indeed a real phenomenon that is useful in understanding various biological processes. In our study of the two brain ageing datasets, we found a number of DV genes that play important roles in normal and pathological neuronal processes. We found that expression variability is generally increasing with age, while decrease in variability with age can also be observed.

Differential variability analysis is often ignored in many gene expression studies because the main aim of these studies is to identify genes that have "significant" changes in mean expression across the study population. However, it is clear that such responses are not sufficient to capture the information in the data. It is important to acknowledge that expression of a gene varies across the population, and this expression variability can change depending on factors such as age and disease. Our previous work showed that genes with decreased variability also tend to have decreased gene-to-gene coexpression in human diseases, which implies that loss of gene expression variability is associated with a loss in gene regulation [10].

Our quantile regression approach is a very powerful tool to assess DV in time-series microarray data, thus opening up the opportunity for a large scale meta-analysis of many microarray datasets to assess the prevalence of DV in human and other organisms, in ageing and diseases. We believe a good understanding of population based gene expression variability is a crucial step towards developing personalized medicine strategies [39,40].

### Extension to analysis of microarray datasets with multiple discrete class labels

While preparing this manuscript, we realized that our quantile regression approach can be extended to identify genes with DE and DV patterns in microarray datasets with discrete class labels. The general concept of fitting and comparing a small number of competing models to a dataset (such as a non-DE model vs. a DE model) can be readily applied to identifying genes with interesting patterns, where these patterns are predefined using biological knowledge and are encapsulated in the model formulation. Here we propose a simple approach to identify genes that have class-dependent DE and DV patterns.

**Figure 8:**
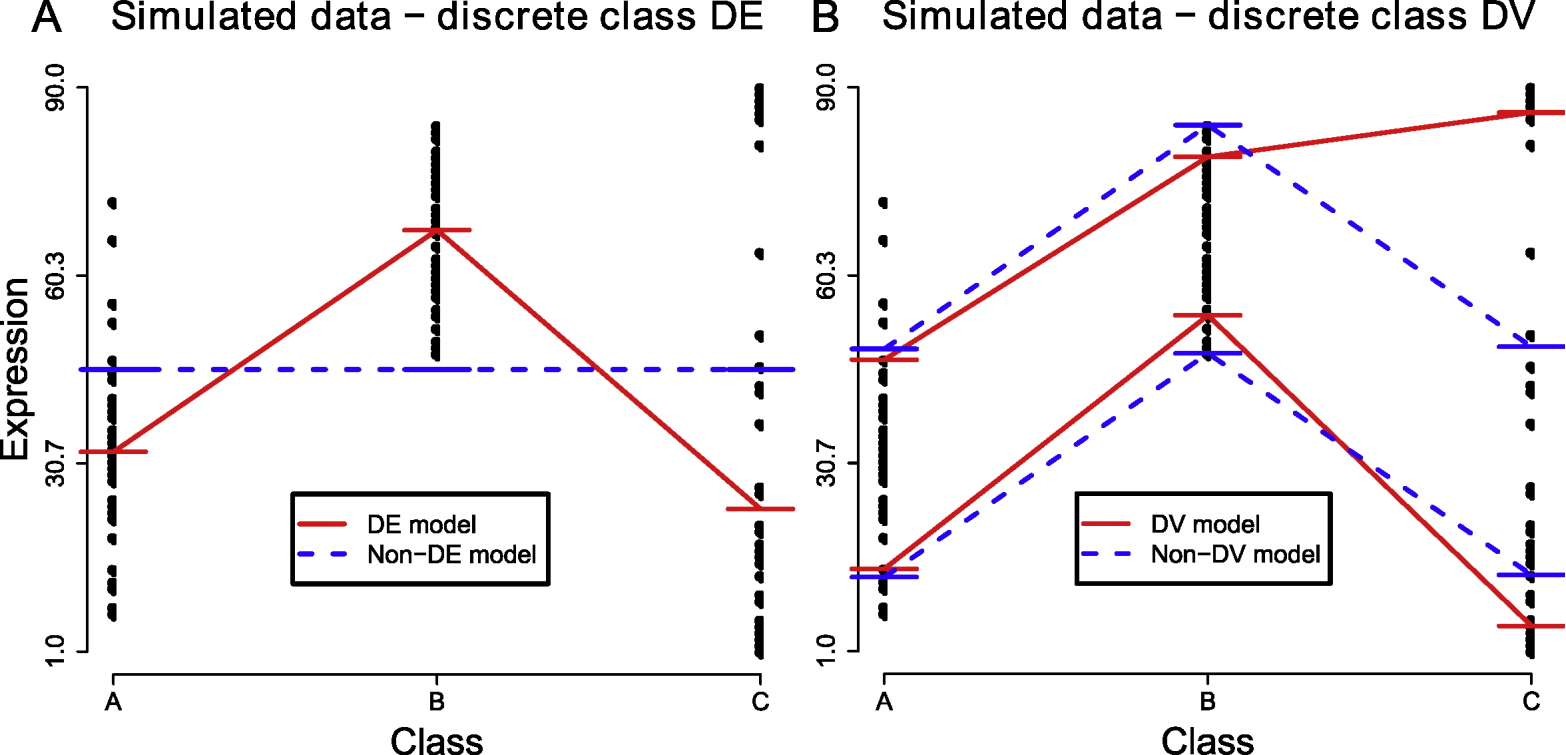
**A proposed approach to identify DE and DV genes in a multi-class microarray dataset using quantile regression**. Genes that are DE across multiple classes of samples can be identified by checking whether the RSAD for fitting a DE model, which specifies one median value per class is much smaller than the RSAD for a simpler model (a non-DE model) which specifies only one median value for all *k* classes. Similarly, we can identify DV genes by checking whether the DV model with 2*k *parameters is much better than the non-DV model with *k *+ 1 parameters, where *k *is the number of distinct classes.

For identifying DE genes, we propose to fit and compare the goodness-of-fit of two models, where one model specifies that the median expression values are the same across multiple classes (the non-DE model), and the second model specifies that the median expression values can differ across multiple classes (the DE model). The non-DE model only requires fitting one parameter — the median value of the data, while the DE model requires fitting *k* parameters where *k *is the number of distinct class labels. If the RSAD of the DE model is much smaller than the RSAD of the non-DE model, based on a predefined threshold, a gene is deemed to be differentially expressed (see Figure 8A for an example).

For identifying genes with DV, one can similarly fit and compare two competing models — the non-DV model and the DV model. The non-DV model specifies that the lower quantile for each class is estimated independently while sharing the same inter-quantile range (the absolute difference between the upper and lower quantiles) among the *k *classes. The DV model specifies that both the lower quantile and the inter-quantile range of each class are independent. If the RSAD of the DV model is much smaller than the RSAD of the non-DV model, based on a predefined threshold estimated as before, a gene is deemed to have a DV pattern (see Figure 8B for an example). It should be noted that the non-DV and DV models here differ only by whether the inter-quantile range is the same across all classes, so the detection of DV should be independent of whether a gene is DE or not. We believe that this proposed approach can overcome the limitation of the simple DV analysis procedure that we described previously [10].

Similar to linear regression, quantile regression techniques are most commonly used in finding 'interesting' trends in time-series data, such as various econometric, social and ecological data [14]. We note that our proposed approach for analyzing multi-class microarray datasets is a novel application of quantile regression technique to analyze non-time series data. Although further research is required to investigate the applicability of our proposal, we show conceptually how quantile regression models may be applied to a broader range of non-time series data analysis problems.

## Conclusion

The main objective of this paper is to present and evaluate a novel approach to discovering genes with various age-dependent expression patterns. Through an extensive simulation study, we show that our quantile regression approach is superior to linear regression based methods in terms of sensitivity and specificity of identifying linear and non-linear DE and DV patterns. We applied our method to two human brain ageing microarray datasets and show that biologically interesting patterns can be discovered.

Further, we propose that our model selection approach to pattern identification can be extended to handle DE and DV discovery tasks in microarray datasets with multiple discrete class labels. Therefore we believe that our approach is an important tool in our quest to understand the nature of gene expression regulation.

## Methods

### Simulation of artificial microarray data

We simulated seven datasets with different noise properties to evaluate the performance of our quantile regression method compared to other linear regression based methods (described in the next subsection) in terms of discovering DE and DV patterns. All simulated datasets contained 3000 genes and 80 samples, and the samples were grouped into 8 groups of 10 samples. Among the 3000 genes, 1000 of them had no age-dependent expression patterns (C), 1000 of them had a linear age-dependent trend (L), and the remaining 1000 genes had a non-linear age-dependent trend (NL). All data values in each artificial dataset were drawn from a normal distribution with mean *μ *and standard deviation *σ*. The *μ *and *σ *of each data point depended on the dataset and the age group to which its sample belonged. In other words, various age-dependent expression patterns were simulated by choosing different *μ *and *σ *for each set of genes at different age groups (see Table 2). Datasets containing outliers were simulated by randomly selecting some data points in the dataset and scaling their value by an arbitrary amount randomly drawn from a uniform distribution *U*(-5, 5).

**Table 2:** Parameters for simulating the seven artificial datasets.

		Sample (in ascending order of age)							
Dataset	Pattern	1-10	11-20	21-30	31-40	41-50	51-60	61-70	71-80
DE, 2	C	4, 2	4, 2	4, 2	4, 2	4, 2	4, 2	4, 2	4, 2
	L	1, 2	2, 2	3, 2	4, 2	5, 2	6, 2	7, 2	8, 2
	NL	1, 2	2, 2	3, 2	4, 2	5, 2	4, 2	3, 2	1, 2

DE, 5	C	4, 5	4, 5	4, 5	4, 5	4, 5	4, 5	4, 5	4, 5
	L	1, 5	2, 5	3, 5	4, 5	5, 5	6, 5	7, 5	8, 5
	NL	1, 5	2, 5	3, 5	4, 5	5, 5	4, 5	3, 5	1, 5

DE, 5+outliers	C	4, 5	4, 5	4, 5	4, 5	4, 5	4, 5	4, 5	4, 5
	L	1, 5	2, 5	3, 5	4, 5	5, 5	6, 5	7, 5	8, 5
	NL	1, 5	2, 5	3, 5	4, 5	5, 5	4, 5	3, 5	1, 5

DE, 9	C	4, 9	4, 9	4, 9	4, 9	4, 9	4, 9	4, 9	4, 9
	L	1, 9	2, 9	3, 9	4, 9	5, 9	6, 9	7, 9	8, 9
	NL	1, 9	2, 9	3, 9	4, 9	5, 9	4, 9	3, 9	1, 9

DE, 9+outliers	C	4, 9	4, 9	4, 9	4, 9	4, 9	4, 9	4, 9	4, 9
	L	1, 9	2, 9	3, 9	4, 9	5, 9	6, 9	7, 9	8, 9
	NL	1, 9	2, 9	3, 9	4, 9	5, 9	4, 9	3, 9	1, 9

DV	C	4, 3	4, 3	4, 3	4, 3	4, 3	4, 3	4, 3	4, 3
	L	4, 1	4, 2	4, 3	4, 4	4, 5	4, 6	4, 7	4, 8
	NL	4, 1	4, 2	4, 3	4, 4	4, 5	4, 3	4, 2	4, 1

DV+outliers	C	4, 3	4, 3	4, 3	4, 3	4, 3	4, 3	4, 3	4, 3
	L	4, 1	4, 2	4, 3	4, 4	4, 5	4, 6	4, 7	4, 8
	NL	4, 1	4, 2	4, 3	4, 4	4, 5	4, 3	4, 2	4, 1

Gene expression values can be simulated by drawing values from a normal distribution with mean *μ *and standard deviation *σ*. The choice of *μ *and *σ *depends on the age group of a sample and the type of pattern each gene is simulating. The complete list of *μ *and *σ *used to simulate the seven artificial microarray datasets are shown here, given as *μ*, *σ *pairs.

### Comparison with linear regression based methods

To provide a baseline for comparison, we also analyzed our simulated datasets with two linear regression based methods for DE and DV patterns discovery. For identifying genes with either linear or non-linear DE patterns, we used a second order linear regression model of the form *f*(*x*) = *a *+ *b*_1_*x *+ *b*_2_*x*^2^. We then independently tested whether *b*_1 _= 0 and *b*_2 _= 0 by performing a *t*-test on each parameter, which gives us two *p*-values: *p*_1 _and *p*_2 _respectively for parameter *b*_1 _and *b*_2_. Given a predefined significance level *α*_*l*_, a gene is classified to have one of the three patterns using the following set of rules:

A second order linear regression model is more commonly known as the quadratic regression model, but we deliberately avoid the this terminology since it may be easily confused with the term quantile regression, also abbreviated as QR. We note that the above linear regression based method is a variant of the quadratic regression method of Liu *et al*. [41].

To identify genes with linear or non-linear DV patterns using a linear regression based method, we use the following two step scheme: (1) Fit the data with a third-order linear regression model (commonly known as a cubic regression model) of the form *f*(*x*) = *a *+ *b*_1_*x *+ *b*_2_*x*^2 ^+ *b*_3_*x*^3^, and obtain the residuals as *r*_*i *_= *y*_*i *_- *f*(*x*_*i*_), then (2) Fit a second order linear model to the absolute residuals, i.e., |*r*| = *a *+ *b*_1_*x + b*_2_*x*^2^, which enables us to calculate a *p*-value for each of *b*_1 _and *b*_2 _(*p*_1 _and *p*_2 _respectively) using *t*-tests. Using the two *p*-values, we have the following rules for determining whether a gene has a 'significant' DV pattern given a significance level *α*_*l*_:

The linear regression model fitting and *p*-value calculation are performed by the function lm in R.

### Construction and interpretation of the ROC curves

A Receiver Operator Characteristic (ROC) curve is a two dimensional plot of two important performance measures of a pattern discovery method — the true positive rate (TPR; or sensitivity) and the false positive rate (FPR; or 1-specificity). A desirable pattern discovery method should achieve a high TPR while maintaining a low FPR. If TPR = FPR for all threshold values, the pattern discovery method is performing just as badly as a random binary classifier that randomly assigns an object into one of the two classes with probability 0.5. Given the true positive (TP), true negative (TN), false positive (FP) and false negative (FN) rates at a given *α*, TPR = TP/(TP+FN) and FPR = FP/(FP+TN).

### Analysis of real datasets

The Colantuoni dataset [6] and the Lu dataset [2] were obtained from the Gene Expression Omnibus (GEO) [42] using accession number GSE11546 and GSE1572 respectively. We used the preprocessed data available for each dataset to enable maximum consistency with the original studies.

We estimated the false discovery rate (FDR) of our procedure in discovering age-dependent patterns in the two real datasets using a randomization procedure. Using the concepts and notation developed by Storey and Tibshirani [43], a false discovery rate given a particular threshold value *α *is the expected proportion of false positives (F) in all positive calls (S), which can be written as

The last approximation can be shown to be valid if the number of genes tested is large [43]. In our estimation, we approximate *E*[*S*(*α*)] to be the number of genes identified to have an age-dependent pattern (either DE or DV) based on the threshold *α*, and we estimate *E*[*F*(*α*)] as the average number of genes identified to have an age-dependent pattern in *m *permuted datasets, by uniformly permuting the age associated with samples without altering the expression data. Since the resulting FDR estimates are relatively stable among permutations, we decided to set *m *= 10 in analyzing both real datasets as we can already obtain a reasonable estimate of FDR, without further computational expenses.

## Competing interests

The authors declare that they have no competing interests.

## Authors' contributions

JWKH conceived, designed and performed the research, analyzed data and wrote the manuscript. MS and JWKH conceived the idea of differential variability in human ageing. MS and CGdR contributed to the biological interpretation of the results. MAC supervised the study, helped design the experiments and critically revised the manuscript. All authors read and approved the final version of the manuscript.

## Note

Other papers from the meeting have been published as part of *BMC Bioinformatics* Volume 10 Supplement 15, 2009: Eighth International Conference on Bioinformatics (InCoB2009): Bioinformatics, available online at http://www.biomedcentral.com/1471-2105/10?issue=S15.

## References

[B1] Hekimi S How genetic analysis tests theories of animal aging Nat Genet 2006 38 985 991 10.1038/ng1881 16941009

[B2] Lu T Pan Y Kao SY Li C Kohane I Chan J Yankner BA Gene regulation and DNA damage in the aging human brain Nature 2004 429 883 891 10.1038/nature02661 15190254

[B3] Volkova M Garg R Dick S Boheler KR Aging-associated changes in cardiac gene expression Cardiovasc Res 2005 66 194 204 10.1016/j.cardiores.2004.11.016 15820188

[B4] Pan F Chiu CH Pulapura S Mehan MR Nunez-Iglesias J Zhang K Kamath K Waterman MS Finch CE Zhou XJ Gene Aging Nexus: a web database and data mining platform for microarray data on aging Nucl Acids Res 2007 35 D756 D759 10.1093/nar/gkl798 17090592 PMC1669755

[B5] Zahn JM Poosala S Owen AB Ingram DK Lustig A Carter A Weeraratna AT Taub DD Gorospe M Mazan-Mamczarz K Lakatta EG Boheler KR Xu X Mattson MP Falco G Ko MSH Schlessinger D Firman J Kummerfeld SK Wood WH Zonderman AB Kim SK Becker KG AGEMAP: A Gene Expression Database for Aging in Mice PLoS Genet 2007 3 e201 10.1371/journal.pgen.0030201 18081424 PMC2098796

[B6] Colantuoni C Hyde TM Mitkus S Joseph A Sartorius L Aguirre C Creswell J Johnson E Deep-Soboslay A Herman MM Lipska BK Weinberger DR Kleinman JE Age-related changes in the expression of schizophrenia susceptibility genes in the human prefrontal cortex Brain Struct Funct 2008 213 255 271 10.1007/s00429-008-0181-5 18470533

[B7] de Magalhães JP Curado J Church GM Meta-analysis of age-related gene expression profiles identifies common signatures of aging Bioinformatics 2009 25 875 881 10.1093/bioinformatics/btp073 19189975 PMC2732303

[B8] Somel M Khaitovich P Bahn S Pääbo S Lachmann M Gene expression becomes heterogeneous with age Curr Biol 2006 16 R359 R360 10.1016/j.cub.2006.04.024 16713941

[B9] Li Z Wright FA Royland J Age-Dependent Variability in Gene Expression in Male Fischer 344 Rat Retina Toxicol Sci 2009 107 281 29 10.1093/toxsci/kfn215 18936298 PMC4796736

[B10] Ho JWK Stefani M dos Remedios CG Charleston MA Differential variability analysis of gene expression and its application to human diseases Bioinformatics 2008 24 13 i390 i398 10.1093/bioinformatics/btn142 18586739 PMC2718620

[B11] Bahar R Hartmann CH Rodriguez KA Denny AD Busuttil RA Dollé ME Calder RB Chisholm GB Pollock BH Klein CA Vijg J Increased cell-to-cell variability in gene expression in ageing mouse heart Nature 2006 441 1011 1014 10.1038/nature04844 16791200

[B12] Bar-Joseph Z Analyzing time-series gene expression data Bioinformatics 2004 20 2493 2503 10.1093/bioinformatics/bth283 15130923

[B13] Androulakis I Yang E Almon RR Analysis of Time-Series Gene Expression Data: Methods, Challenges, and Opportunities Annu Rev Biomed Eng 2007 9 205 228 10.1146/annurev.bioeng.9.060906.151904 17341157 PMC4181347

[B14] Koenker R Quantile Regression 2005 Cambridge University Press, New York, USA

[B15] Koenker R Bassett G Regression Quantiles Econometrica 1978 46 33 50 10.2307/1913643

[B16] Koenker R Hallock KF Quantile Regression J Econ Perspect 2001 15 143 156

[B17] Cade BS Terrell JW Schroeder RL Estimating effects of limiting factors with regression quantiles Ecology 1999 80 311 323

[B18] Cade BS Noon BR A gentle introduction to quantile regression for ecologists Front Ecol Environ 2003 1 412 420

[B19] Eilers PH de Menezes RX Quantile smoothing of array CGH data Bioinformatics 2005 21 1146 1153 10.1093/bioinformatics/bti148 15572474

[B20] Li Y Zhu J Analysis of array CGH data for cancer studies using fused quantile regression Bioinformatics 2007 23 2470 2476 10.1093/bioinformatics/btm364 17644559

[B21] Huang L Zhu W Saunders CP MacLeod JN Zhou M Stromberg AJ Bathke AC A novel application of quantile regression for identification of biomarkers exemplified by equine cartilage microarray data BMC Bioinformatics 2008 9 300 10.1186/1471-2105-9-300 18597687 PMC2474617

[B22] Cho H Kim Y Jung HJ Lee SW Lee JW an R package for outlier detection using quantile regression on mass spectrometry data Bioinformatics 2008 24 882 884 10.1093/bioinformatics/btn012 18187441

[B23] Burnham KP Anderson DR Model Selection and Multimodel inference: A practical information-theoretic approach (2nd) 2002 Springer

[B24] Newell-Litwa K Salazar G Smith Y Faundez V Roles of BLOC-1 and adaptor protein-3 complexes in cargo sorting to synaptic vesicles Mol Biol Cell 2009 20 1441 1453 10.1091/mbc.E08-05-0456 19144828 PMC2649275

[B25] Murphy S Krainock R Tham M Neuregulin signaling via erbB receptor assemblies in the nervous system Mol Neurobiol 2002 25 67 77 10.1385/MN:25:1:067 11890458

[B26] Park D Reuter-Lorenz P The adaptive brain: aging and neurocognitive scaffolding Annu Rev Psychol 2009 60 173 196 10.1146/annurev.psych.59.103006.093656 19035823 PMC3359129

[B27] Sanna B Brandt E Kaiser R Pfluger P Witt S Kimball T van Rooij E De Windt L Rothenberg M Tschop M Benoit S Molkentin J Modulatory calcineurin-interacting proteins 1 and 2 function as calcineurin facilitators in vivo Proc Natl Acad Sci USA 2006 103 7327 7332 10.1073/pnas.0509340103 16648267 PMC1464340

[B28] Enderlin V Pallet V Alfos S Dargelos E Jaffard R Garcin H Higueret P Age-related decreases in mRNA for brain nuclear receptors and target genes are reversed by retinoic acid treatment Neurosci Lett 1997 229 125 129 10.1016/S0304-3940(97)00424-2 9223607

[B29] Mons N Enderlin V Jaffard R Higueret P Selective age-related changes in the PKC-sensitive, calmodulin-binding protein, neurogranin, in the mouse brain J Neurochem 2001 79 859 867 10.1046/j.1471-4159.2001.00646.x 11723178

[B30] Pak J Huang F Li J Balschun D Reymann K Chiang C Westphal H Huang K Involvement of neurogranin in the modulation of calcium/calmodulin-dependent protein kinase II, synaptic plasticity, and spatial learning: A study with knockout mice Proc Natl Acad Sci USA 2000 97 11232 11237 10.1073/pnas.210184697 11016969 PMC17183

[B31] Miyakawa T Yared E Pak J Huang F Huang K Crawley J Neurogranin null mutant mice display performance deficits on spatial learning tasks with anxiety related components Hippocampus 2001 11 763 775 10.1002/hipo.1092 11811671

[B32] Huang K Huang F Jäger T Li J Reymann K Balschun D Neurogranin/RC3 enhances long-term potentiation and learning by promoting calcium-mediated signaling J Neurosci 2004 24 10660 10669 10.1523/JNEUROSCI.2213-04.2004 15564582 PMC6730132

[B33] Miller K Verma A Snyder S Ross C Localization of an endoplasmic reticulum calcium ATPase mRNA in rat brain by in situ hybridization Neuroscience 1991 43 1 9 10.1016/0306-4522(91)90410-P 1833665

[B34] Zhao C Slevin J Whiteheart S Cellular functions of NSF: not just SNAPs and SNAREs FEBS Lett 2007 581 2140 2149 10.1016/j.febslet.2007.03.032 17397838 PMC1948069

[B35] Yepes M Lawrence D a selective inhibitor of tissue-type plasminogen activator in the central nervous system Thromb Haemost 2004 91 457 464 14983220 10.1160/TH03-12-0766

[B36] Chamberlin T The Method of Multiple Working Hypotheses Science 1890 15 92 [(*a reprint is published in Science *148, 754-759)]. 10.1126/science.ns-15.366.92 17782687

[B37] Vieth E Fitting piecewise linear regression functions to biological responses J Appl Physiol 1989 67 390 396 2759968 10.1152/jappl.1989.67.1.390

[B38] Chappell R Fitting Bent Lines to Data, with Applications to Allometry J Theor Biol 1989 138 235 256 10.1016/S0022-5193(89)80141-9 2607772

[B39] Ahn AC Tewari M Poon CS Phillips RS The Clinical Applications of a Systems Approach PLoS Med 2006 3 e209 10.1371/journal.pmed.0030209 16683861 PMC1459481

[B40] Deisboeck TS Personalizing medicine: a systems biology perspective Mol Syst Biol 2009 5 249 10.1038/msb.2009.8 19293829 PMC2671924

[B41] Liu H Tarima S Borders AS Getchell TV Getchell ML Stromberg AJ Quadratic regression analysis for gene discovery and pattern recognition for non-cyclic short time-course microarray experiments BMC Bioinformatics 2005 6 106 10.1186/1471-2105-6-106 15850479 PMC1127068

[B42] Edgar R Domrachev M Lash AE NCBI gene expression and hybridization array data repository Nucelic Acids Res 2002 30 207 210 10.1093/nar/30.1.207 PMC99122 11752295

[B43] Storey JD Tibshirani R Statistical significance for genomewide studies Proc Natl Acad Sci USA 2003 100 9440 9445 10.1073/pnas.1530509100 12883005 PMC170937

